# Simultaneous Motor and Visual Intraoperative Neuromonitoring in Asleep Parietal Lobe Surgery: Dual Strip Technique

**DOI:** 10.3390/jpm12091478

**Published:** 2022-09-09

**Authors:** Devika Rajashekar, Jose Pedro Lavrador, Prajwal Ghimire, Hannah Keeble, Lauren Harris, Noemia Pereira, Sabina Patel, Ahmad Beyh, Richard Gullan, Keyoumars Ashkan, Ranjeev Bhangoo, Francesco Vergani

**Affiliations:** 1 Department of Neurosurgery, King’s College Hospital NHS Foundation Trust, London SE5 9RS, UK; 2 Inomed Neurocare Ltd., London W6 0NB, UK; 3 Neurosurgery Department, Queen’s Hospital, Barking, Havering and Redbridge University Hospitals NHS Trust, London RM7 0AG, UK; 4 NatBrainLab, Neuroimaging Department, Institute of Psychiatry, Psychology and Neuroscience, King’s College, London SE5 8AF, UK

**Keywords:** corticospinal tract, optic radiations, tractography, transcranial magnetic stimulation, subdural strip electrodes, intra-operative neuro-monitoring, parietal lobe

## Abstract

**Background**: The role played by the non-dominant parietal lobe in motor cognition, attention and spatial awareness networks has potentiated the use of awake surgery. When this is not feasible, asleep monitoring and mapping techniques should be used to achieve an onco-functional balance. **Objective**: This study aims to assess the feasibility of a dual-strip method to obtain direct cortical stimulation for continuous real-time cortical monitoring and subcortical mapping of motor and visual pathways simultaneously in parietal lobe tumour surgery. **Methods**: Single-centre prospective study between 19 May–20 November of patients with intrinsic non-dominant parietal-lobe tumours. Two subdural strips were used to simultaneously map and monitor motor and visual pathways. **Results**: Fifteen patients were included. With regards to motor function, a large proportion of patients had abnormal interhemispheric resting motor threshold ratio (iRMTr) (71.4%), abnormal Cortical Excitability Score (CES) (85.7%), close distance to the corticospinal tract—Lesion-To-Tract Distance (LTD)—4.2 mm, Cavity-To-Tract Distance (CTD)—7 mm and intraoperative subcortical distance—6.4 mm. Concerning visual function, the LTD and CTD for optic radiations (OR) were 0.5 mm and 3.4 mm, respectively; the mean intensity for positive subcortical stimulation of OR was 12 mA ± 2.3 mA and 5/6 patients with deterioration of VEPs > 50% had persistent hemianopia and transgression of ORs. Twelve patients remained stable, one patient had a de-novo transitory hemiparesis, and two showed improvements in motor symptoms. A higher iRMTr for lower limbs was related with a worse motor outcome (*p* = 0.013) and a longer CTD to OR was directly related with a better visual outcome (*p* = 0.041). At 2 weeks after hospital discharge, all patients were ambulatory at home, and all proceeded to have oncological treatment. **Conclusion**: We propose motor and visual function boundaries for asleep surgery of intrinsic non-dominant parietal tumours. Pre-operative abnormal cortical excitability of the motor cortex, deterioration of the VEP recordings and CTD < 2 mm from the OR were related to poorer outcomes.

## 1. Introduction 

The challenge of neuro-oncology surgery is finding the balance between maximal safe resection and function preservation [1]. This is enabled by integrating pre-operative surgical mapping (cortical and subcortical), with intraoperative mapping and monitoring, to delineate functional resection boundaries [2].

The parietal lobe has a role in language (mainly dominant hemisphere) [1], spatial awareness [3], motor cognition [4], and attention [5]. For these reasons, awake parietal surgery is increasingly performed [6] if possible. Large tumours with mass effect, decreased consciousness, poor cooperation, comorbidities, neuropsychological factors, and patient preference are contraindications [7]. Lack of intraoperative communication (due to dysphasia and antiepileptics), seizures, emotional intolerance, airway management, and electrolyte imbalance are factors associated with failed awake craniotomy, that correlate with poorer outcomes (language and length of stay) [8,9,10,11]. Failed awake surgery is related to a lesser extent of resection [8]. 

Motor and visual functions are at risk during parietal lobe surgery. The corticospinal tract (CST) extending from the primary motor cortex to the corona radiata and internal capsule anteriorly, and the optic radiations (ORs) extending from the lateral geniculate body via the stratum sagittale to the primary visual cortex laterally and inferiorly in an antero-posterior direction, are significant subcortical functional boundaries. Monitoring and mapping of motor functions improves surgical outcomes [12]. 

This study assesses the feasibility of a dual-strip method to obtain direct cortical stimulation, to gain continuous, real-time cortical monitoring and subcortical mapping of motor and visual pathways simultaneously. We propose this method to identify the motor and visual functions as onco-functional limits for parietal surgery in patient’s ineligible for awake surgery.

## 2. Methods

This is a prospective single-centre cohort study from May 2019 to November 2020 at a tertiary neuro-oncology hospital. The inclusion criteria were age ≥ 18, intra-axial, parietal lesions, informed consent for intra-operative neuro-monitoring (IONM), and preoperative surgical mapping (navigated transcranial magnetic stimulation (nTMS) and/or tractography). Ethics committee approval was not sought as the methodology of this study did not alter the surgical methods, and instead only added as operative adjuncts. The exclusion criteria were emergency admission and unavailability of IONM. 

All patients underwent image-guided surgery with neuro-navigation (StealthStation S8 Medtronic). Augmented reality gained 3D visualisation of the tumour and tracts intra-operatively (object-brain overlay technique). 

Post-operatively, all patients received a pre- and post-gadolinium Magnetic Resonance Imaging (MRI) scan (within 48 h). Gross total resection (GTR) was determined by no contrast enhancement (reported by a consultant neuroradiologists), and a subtotal resection (STR) by residual enhancement. The Cavity-to-Tract Distance (CTD) was calculated by merging the postoperative T1-weighted with Gadolinium MRI with the preoperative tractography (affine co-registration) as previously described by other authors [13].

## 3. Intraoperative Neuromonitoring

### 3.1. Monitoring and Mapping the CST

After intubation of the patient, electrodes were placed in muscle groups of the hemibody contralateral to the tumour (face—*orbicularis oris* and tongue; upper limb—*deltoid*, *brachioradialis*, *flexor carpi ulnaris*, *first dorsal interossei*, *abductor digiti minimi*, *abductor pollicis brevis*; lower limb—quadriceps, *tibialis anterior* and *abductor hallucis*), to detect motor responses. Bilateral electrodes were placed in the extremities to act as controls. Scalp corkscrew electrodes facilitated intermittent transcranial monitoring of motor evoked potentials (MEPs), and somatosensory evoked potentials (SSEPs). A subdural strip electrode was placed over the motor cortex, identified anatomically, with tractography of the CST and motor nTMS preoperative mapping, using neuro-navigation. One functional area was targeted—most commonly the area of the upper limb given its wider representation—as the strip was used for monitoring only (no tumour was infiltrating the primary motor cortex) and the elected functional area was considered a surrogate for the whole CST when it was approached at the level of the corona radiata or internal capsule. This facilitated continuous direct cortical stimulation (DCS), monitoring the stability of MEPs, and the integrity of the CST. Constant-current technology delivered high frequency stimulation using the train-of-5 anodal square pulse technique comprised of 500 µs pulse width, inter-stimulus-interval of 2–4 ms, and 1–25 mA was utilized for the cortical mapping and monitoring with the strip electrode and monopolar probe; a similar technique with cathodal pulses mapped the CST subcortically with a monopolar suction probe (Inomed Medizintechnik GmbH, Emmendingen, Germany) (Supplementary Materials).

### 3.2. Monitoring and Mapping Optic Radiations

To record Visual Evoked Potentials (VEPs), the eyes were stimulated using light-emitting diode (LED) goggles (Inomed Medizintechnik GmbH, Dublin, Ireland) at 14,000–20,000 Ix. Corkscrew electrodes placed over the bilateral occipital lobes and referenced to electrodes placed over the mastoids and cranial midline, obtained intermittent VEPs from scalp recordings (SR) [14]. To obtain VEPs from continuous direct cortical recordings (DCR), a second subdural strip electrode was placed over the visual cortex (calcarine fissure) and referenced to a corkscrew placed on the mastoid or the cranial midline. After gaining access to the midline, the strip was placed perpendicular to the calcarine fissure, and its position was confirmed under direct vision or with neuro-navigation and/or ultrasound. This monitored and mapped optic radiations. Whilst monitoring VEPs, a phase reversal of the VEPs were identified across the calcarine fissure. A ball tip bipolar fork probe with 8 mm between the poles was used to stimulate subcortical tissue intra-operatively with a biphasic pulse form, 1–4 Hz, and current dependent on proximity to the optic radiations (max 20 mA). P2 and N3 peaks (International Society for Clinical Electrophysiology of Vision (ISCEV) standards) were measured at baseline, and during debulking [15] (Supplementary Materials).

For both motor and visual mapping and monitoring, changes in the amplitude and latency of the waves acted as warning signs for the subdural strips to be repositioned. Only after movement of the strip was ruled out and optimal position verified according to preoperative mapping techniques (tractography and nTMS) and anatomical landmarks (hand knob and calcarine sulcus), the changes in the amplitude and latency of the waves were considered true monitoring warning signs.

### 3.3. Tractography

Pre-operatively, diffusion tensor imaging (DTI) and T1-post-gadolinium MRI scans were uploaded to StealthViz^®^ (Medtronic) to delineate the CST and optic radiations. The closest distance (millimeters) between tract and tumour was calculated (zero if direct contact). Post-operative MRI was merged with the pre-operative tractography, and two people independently measured the closest distance of each tract to the resection cavity, using the mean. 

In order to visualise the ipsilateral CST, a region of interest (ROI) was placed over the pre-central gyrus and a second ROI on the midbrain. The FA start value of 0.18 was used along with a maximal directional change of 45°. With knowledge of the tract anatomy, manual dissection was used to remove spurious tracts. For the ipsilateral optic radiations, an ROI was placed over the lateral geniculate body and a second ROI over the visual cortex. Once again, the FA start value of 0.18 was used, with a maximal directional change of 60°. Fibres that were not following the anatomical pathway for these tracts were dissected appropriately.

### 3.4. Navigated Transcranial Magnetic Stimulation (nTMS) 

nTMS uses a high-precision coil, neuronavigation, and appropriate software to deliver biphasic magnetic stimulation to the cortex. A single-pulse nTMS applied to the primary motor cortex, generates a muscle output that is recorded via continuous EMG.

nTMS was performed as a non-invasive adjunct for preoperative motor mapping. A T1 weighted post contrast MRI sequence for each patient was uploaded onto the Nexstim© (Helsinki, Finland) TMS hardware to enable accurate mapping of the motor cortex and collection of data on the resting motor threshold (RMT), latency, amplitude, interhemispheric resting motor threshold ratio (iRMTr), and the cortical excitability score (CES).

Continuous electromyography was used to monitor motor evoked potentials (MEPs) of the *abductor policis brevis* (APB), *first dorsal interossus* (FDI), and the *abductor digiti minimi* (ADM) in both upper limbs, as well as the *tibialis anterior* (TA) and *extensor hallucis longus* (EHL) in both lower limbs. Single pulse stimulation was applied at 1 hz to both hemispheres at rest to identify the motor areas and ascertain the RMTs. Positive muscle responses were defined as MEPs greater than 50 µV. Once determined, a final motor map was generated over the hemisphere of interest at 105% of the RMT. 

The iRMTr was calculated as a ratio of the RMTs between the limbs in both hemispheres and was considered to be pathological if there was a difference of more than 10%. The Cortical Excitability Score (CES) was calculated and defined as the number of pathological iRMTs recorded: 0 (no pathological iRMTr present); 1 (only one pathological iRMTr present, either for the upper or lower limb); and 2 (when both upper and lower limb demonstrated a pathological iRMTr) (Supplementary Materials).

The motor maps generated were exported as DICOM files and used for intraoperative augmented reality and navigation.

### 3.5. Surgical Adjuncts

5-Aminolevulinic Acid was used in suspected high-grade gliomas using the BLUE 400 florescence filter (KINEVO^®^ 900, Zeiss, Oberkochen, Germany). Intraoperative Ultrasound (MyLab™Eight Ultrasound, Esaote, Genoa, Italy) determined the position of the subdural strip directed to the calcarine sulcus. Tubular-retractor systems (BrainPath^®^, NICO, Indianapolis, IN, USA and The ViewSite™ Brain Access System, Vycor Medical, Boca Raton, FL, USA) accessed deep lesions, allowing minimally invasive parafascicular surgery (MIPS). 

### 3.6. Schematic Illustrative Figure 

Pre-processed diffusion-weighted MRI data were retrieved for a young adult male from the Human Connectome Project (www.humanconnectome.org (accessed on 8 June 2022)). Diffusion tensor modelling and tractography were computed in StarTrack (www.mr-startrack.com (accessed on 2 July 2022)) according to the following criteria: minimum fractional anisotropy threshold = 0.2; step size = 0.5 mm; maximum angle threshold = 30°. The optic radiations and corticospinal tract were manually dissected in TrackVis (www.trackvis.org (accessed on 3 July 2022)). The CST was dissected using an axial waypoint ROI at the level of the pons and another to intersect terminations in sensorimotor cortex. The optic radiation was dissected using a termination ROI at the level of the lateral geniculate nucleus and a coronal waypoint ROI at the level of the occipital lobe. The dissected tracts were aligned to the MNI152 brain template for final display within the brain surface in SurfIce (www.nitrc.org/projects/surfice (accessed on 1 July 2022)). The locations of each patient’s response points for the CST and OR were manually estimated on the MNI152 T1-weighted image, and the resulting coordinates were imported into the final display for illustrative purposes (Supplementary Materials).

### 3.7. Statistical Analysis

STATA 13.1© (StataCorp, College Station, TX, USA) was used. Linear regression assessed the lesion-to-tract distance (LTD), cavity-to-tract distance (CTD), intraoperative distance to CST, and iRMTrs in the motor and visual outcomes. Ordered logistic regression assessed the CES in motor outcome. *p* < 0.05 was considered statistically significant.

## 4. Results

### 4.1. Patient Characteristics

Fifteen patients (11 males, 4 females) with parietal lesions were included, with a median age of 57 (range 23–77). Thirteen underwent primary resection, and two for recurrence. Ultrasound was used in six. Two underwent MIPS. Six lesions were located within the superior parietal lobule, five inferior parietal lobule, and three in both. One patient had a lesion in the transition between the corona radiata and the internal capsule with no cortical expression. Glioblastoma (GBM) was diagnosed in 13 (including two recurrences), with nine MGMT methylated and two isocitrate dehydrogenase-1 (IDH-1) mutant. One had a breast metastasis, and one a pilocytic astrocytoma (Table 1). 

### 4.2. Intraoperative Neuromonitoring

The size of the craniotomy was not related to the need for inserting a subdural strip electrode. The average craniotomy size was 32.0 cm^2^ with a standard deviation of 23.9 (Table 1). The average duration of monitoring was 218 min (range 160–302).

All patients had stable VEPs and MEPs at the beginning of the monitoring (once the strips were placed over primary motor and visual cortices). No complications were verified during the placement of the strip electrodes, in particular bleeding. Moreover, no complications related with continuous monitoring (in particular, seizures) occurred. The mean cortical resting motor threshold with monopolar high-frequency stimulation was 7.5 ± 1.23 mA. Continuous subcortical CST stimulation was performed during the resection, and the mean minimal amplitude of stimulation at end of resection was 6.4 mA (min 3 mA; max 12 mA). Transcranial and continuous MEPs from DCS remained stable. Abnormal iRMTr for the upper limbs was potentially related with longer intraoperative distance to the CST, although not significant (*p* = 0.054). LTD and iRMTrLL were not significantly related with the intraoperative distance to the CST.

**Figure 1:**
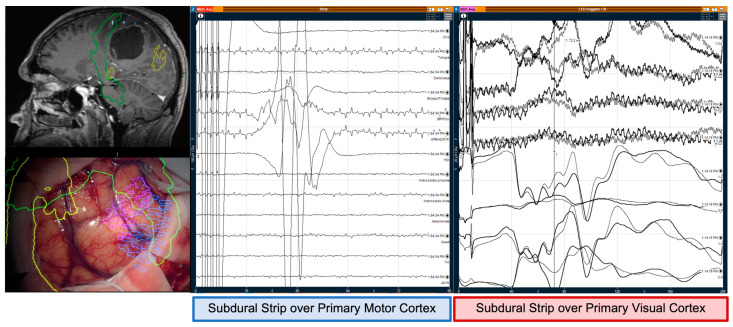
Example of a craniotomy for tumour resection with simultaneously motor and optic radiations’ mapping. The recordings from the subdural strip over primary motor cortex (**left**) show positive motor evoked responses from the right bicep/tricep, brachioradialis/flexor carpi ulnaris, abductor digiti minimi/ abductor pollicis brevis, and first dorsal interossei at 100 µV amplitude and a latency of 36 ms, from direct cortical stimulation using the strip electrode with 5 anodal pulses, 500 µs pulse width, at 10 mA, with software filters of 30–1500 Hz. The recordings from the subdural strip over primary visual cortex (**right**) show visual evoked potentials recorded using the direct cortically placed strip electrode over the calcarine fissure, from simultaneous stimulation of the bilateral eyes through LED goggles placed over the eyelids at 16,000 lx intensity and 3.1 Hz, using software filters of 10–300 Hz. Artefact can be observed on the VEP strip electrode recording channels referenced to Cz’ scalp electrode (top 4 channels with green arrows). The bottom 4 channels observed VEP waveform responses referenced to other channels on the same strip electrode, with the strongest and largest amplitudes seen on contact 1 referenced to contacts 2 and 4 (blue arrows) at 20 µV, P2 peak at ~90 ms, N3 at ~98 ms, and P3 at ~120 ms however despite filters applied, peaks N1, P2, and N2 are difficult to identify due to external artefact.

The optic radiations were mapped subcortically in 13 patients, with a mean intensity of 12 mA ± 2.3 mA. Six patients (5 with previous hemianopia and one with no visual deficit) had deterioration of the VEPs superior to 50% in both amplitude and latency of the evoked potentials. Five had persistent post-operative hemianopia—which correlated with intraoperative transgression of the optic radiations as per preoperative tractography—and one had no visual changes—optic radiations preserved according to preoperative tractography despite the abovementioned reduction in the VEPs (Figure 1). 

**Figure 2:**
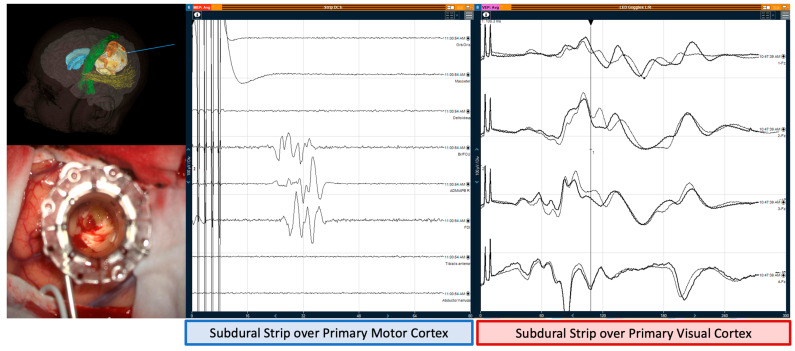
Example of a minimally invasive parafascicular approach (MIPS) for tumour. The recordings from the subdural strip over primary motor cortex (**left**) show positive motor evoked responses from the right brachioradialis/flexor carpi ulnaris, abductor digiti minimi/ abductor pollicis brevis, and first dorsal interossei at 100 µV amplitude and a latency of ~30 ms, from direct cortical stimulation using the strip electrode with 5 anodal pulses, 500 µs pulse width, at 8 mA, with software filters of 30–2000 Hz. The recordings from the subdural strip over primary visual cortex (**right**) show visual evoked potentials recorded using the direct cortically placed strip electrode over the calcarine fissure, from simultaneous stimulation of the bilateral eyes through LED goggles placed over the eyelids at 10,000 lx intensity and 3.1 Hz, using software filters of 10–500 Hz. All four contacts of this strip electrode were referenced to the Fz scalp electrode placed on the left mastoid. The VEPs are observed at 100 µV amplitude, latencies of each peak are seen at N1 ~50 ms, P2 ~60 ms, N3 ~68 ms, P2 ~100 ms, N3 at ~115, and P3 at ~135 ms.

In MIPS cases, there was no change in both MEPs and VEPs before, during, and after the insertion of retractor (Figure 2).

### 4.3. Clinical Outcome

GTR was achieved in 80% (*n* = 12). 

Eleven patients had no motor deficit pre- or post-operatively. Four had a pre-operative contralateral hemiparesis, with two resolving post-operatively. One patient’s weakness improved from Medical Research Council (MRC) grade 3 to grade 4, and one had no change. One had a new contralateral hemiparesis (3/5), which resolved at 3-months.

Nine patients had persistence of pre-operative contralateral homonymous hemianopia. Six had no visual deficits pre- or post-operatively. 

Five developed de novo visual-spatial neglect which they recover at 3-months. There was no surgery related mortality (at 30 days). Nine were discharged home and six (two motor deficit, four de novo visual-spatial neglect) to a step-down neuro-rehabilitation unit for 2 weeks for perioperative intensive rehabilitation before proceeding with ambulatory oncological treatment. At 2 weeks after hospital discharge, all patients were ambulatory at home. All included patients proceed on having oncological treatment after surgery.

### 4.4. Predictive Factors for Clinical Outcome

nTMS was completed for motor movements in seven patients. The pre-operative findings were confirmed intraoperatively with directly cortical stimulation. 6/7 patients had abnormal cortical excitability—six with abnormal interhemispheric RMT ratio (two CES of 1, four CES of 2). 

**Figure 3:**
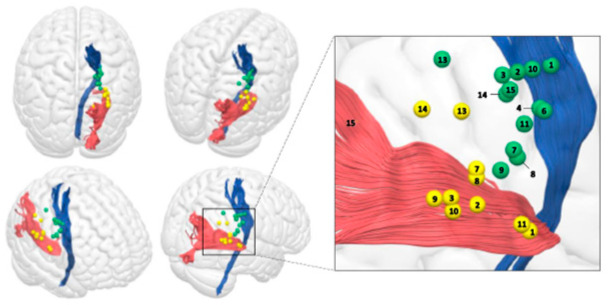
Schematic representation of the positive subcortical positive responses for optic radiations and corticospinal tract. Each number corresponds to the number ID of the patient in Table 1. *Blue*: Corticospinal tract. *Red*: Optic Radiations. *Yellow spheres*: Positive subcortical responses for optic radiations with high frequency bipolar stimulation. *Green spheres*: Positive subcortical responses for the corticospinal tract with high frequency monopolar stimulation.

13 patients had pre-operative tractography (two not possible due to clinical urgency). Pre-operatively, the mean distance of the CST to the tumour was 4.2 mm (range 0–18.1 mm), with four tumours in direct contact (Figure 3). Post-operatively, the mean distance of the resection cavity to the CST was 7 mm (range 0–22.6 mm). Three had the CST in direct contact with the cavity. Pre-operatively, the mean distance of the tumour to the optic radiations was 0.5 mm. Six had optic radiations in direct contact with the lesion, and two tumours had obliterated the optic radiations, correlating with hemianopia pre-operatively. Post-operatively, the mean distance between radiations and the cavity was 3.4 mm (range 0–16.9 mm). Four had tracts in contact with, or within, the resection cavity. The increase in distance is explained by incomplete resection in three, and location of cystic components in two; with significant distortion without invasion of the CST that reverted upon cyst drainage. 

**Figure 4:**
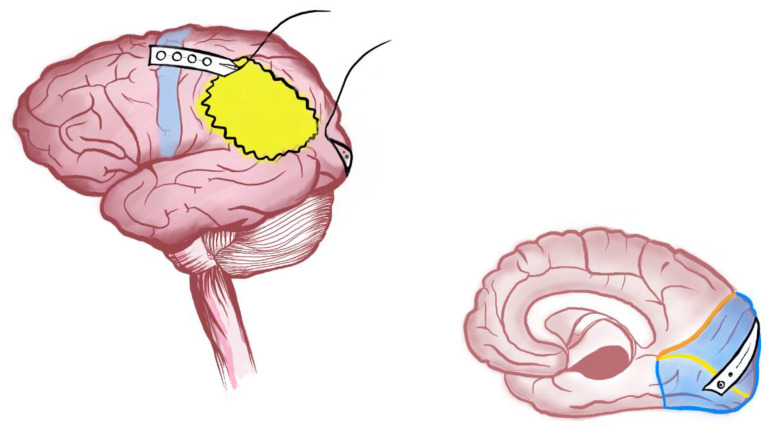
Illustration of Dual Strip technique with strip placed over the motor cortex (*Blue*) (for continuous mapping of CST) and occipital cortex (continuous mapping of VEPs) in the patient with parietal tumour (*Yellow*).

An inverse correlation between the iRMTr and the motor outcome function was statistically significant (*p* = 0.013). No other cortical excitability measures were significant. When the LTD is compared with the motor and visual outcomes, there was no statistical significance (motor—*p* = 0.877; visual—*p* = 0.585). The CTD was not related with motor outcome (*p* = 0.211) but was related to visual outcome (*p* = 0.041)—longer distance in patients with no visual deficit. 5/6 with postoperative hemianopia had CTD ≤ 1 mm, and all with visual deficit had a CTD < 2 mm (only 1/7 with CTD < 2 mm had no postoperative deficit) (Figure 3 and Figure 4).

The impact of the intraoperative minimal distance to the CST and the postoperative motor function was not statistically significant (mean minimal positive subcortical stimulation: post-operative motor deterioration—6 mA; post-operative stable neurology—6.8 ± 0.73; post-operative improvement of motor function—5.5 ± 1.5, *p* = 0.694).

### 4.5. Complications

One patient had meningitis, making a good recovery. 

## 5. Discussion 

This study demonstrates the safety and feasibility of continuous, simultaneous monitoring and mapping of motor and visual function during parietal surgery using two subdural strip electrodes. This was reproduced in highly eloquent motor and visual tumours considering the abnormal motor cortical excitability. To the best of our knowledge, this is the first study that uses subdural strip electrodes to monitor and map MEPs and VEPs simultaneously. The use of integrated neuro-navigation, allowed for strip electrodes to be placed along unexposed cortical surfaces, alleviating the need for large craniotomies. 

The use of subdural strip electrodes for recording of MEPs is a well-established technique for location of motor cortex [16,17]. Monitoring is a useful predictor of deficits, but its value is limited, as signal alterations can be irreversible in 40% [17]. We maintained stable recordings of MEPs in fourteen patients throughout surgery, correlating with functional outcomes. The patient with a de novo transitory hemiparesis had redo surgery for a GBM, with a lesion-to-CST distance of 0 mm (infiltration of the CST) and abnormal cortical excitability; with a high-risk motor eloquent lesion according to pre-operative motor risk stratification scores [2,18]. 

The most recognized method of continuous monitoring of VEPs is via transcranial recording with corkscrew scalp electrodes. Whilst these have shown good results, there is dispute about their correlation with post-operative deficits [19,20,21,22,23], due to low spatial resolution, and the effect of anesthetic agents and brain manipulation on signal reproducibility [24]. Subdural strip electrodes use DCRs to enable cortical and subcortical mapping, to improve the accuracy of signals, and signal to noise ratio [25]. Subdural electrode recordings achieved adequate spatial resolution and intensity of response [26]. Nevertheless, the close relationship between the OR and the inferior fronto-occipital fasciculus (IFOF) in the stratum sagittale as well as their occipital terminations must be acknowledged. Recently, it has been proposed a dual system organization of the stratum sagittale with a core and a peripheral system where both OR and IFOF below to the same core system [27]. 

The current stimulation methods may not have the specificity required to distinguish between the stimulation of these two tracts. Nevertheless, the injury of one tract may increase the risk of injury of other tracts in the same system. Therefore, a positive subcortical-cortical evoked potential should be considered significant. Moreover, if it is elicited in the same place where it was recorded during flashlight stimulation, that would increase the probability of involvement of the OR. 

In this study, patients with previous visual field deficits did not recover after surgery even though we were able to preserve the visual fields in patients with no previous visual deficit. We believe this is related with the infiltrative and aggressive nature of the tumours in most of the included patients (13/15 patients had a diagnosis of WHO Grade 4 Glioblastoma) allied to the fact that patients consented for resection of visual eloquent tumour if the optic radiations were demonstrably infiltrated at the time of surgery (either microscopic or due to 5-ALA positive tissue). This is the main reason why this study is not focused on the potential for preservation of the visual fields with this technique but instead the feasibility and the correlation of the intraoperative findings with the clinical outcomes. 

Abnormal cortical excitability for the lower limbs, as shown by the iRMTrLL, and the distance from the surgical cavity to the optic radiation as per preoperative tractography, as shown by CTD < 2 mm, proved significant. The LTD and CTD analysis did not correlate with the motor outcome. Even though different thresholds are reported in the literature (LTD < 8 mm or LTD < 12 mm) [18,28], those are consistently reviewed intraoperatively by the IONM data, particularly the stability of the continuous MEPs from the subdural strip and the CTD. The intraoperative distance to the CST, calculated with the 1 mm = 1 mA rule, did not correlate with motor outcome. This is explained by the integration of pre-operative and intraoperative mapping data to minimize motor deficits [2]. There is a suggestion of a longer intraoperative distance to the CST in patients where the iRMTr for the upper limb was abnormal. This reflects the understanding of abnormal cortical excitability of the motor cortex as an initial step for motor injury, which requires a more cautious resection towards the M1-CST complex [18,29,30]. For visual outcome, the significance of the CTD supports a need for better intraoperative mapping techniques, though the subdural strip proved to be reliable and predictive of outcome, as a deterioration of the recordings related to postoperative deficit.

Two disadvantages of subdural strip electrodes were the potential for the strips to become displaced (following large debulking), and difficulty accessing the midline in lateral and/or inferior tumours. Feedback from the neurophysiologist, and securing the electrodes prior to debulking, can mitigate this risk. 

The study of complications associated with subdural strip electrode placement has been done largely in the context of epilepsy surgery. Two studies report the rates of subdural haematomas being low which is in-keeping with our experience (*n* = 0). Fountas et al., report a combined rate of bleeding for strip and grid electrodes of 1.1%, whilst Joswig et al., report rates of 1.4% for subdural strip electrodes alone [31,32]. 

Limitations include the fact that this is a single-centre study with a small sample. This study proves the feasibility and describes the pre- and intra-operative technique. To minimize the subjectivity of tract dissections and distances assessed, these were performed independently by two people. The technique used to assess the CTD was previously validated in the literature [13]. We are aware that this method is dependent on co-registration, and it is not new diffusion data for de novo postoperative tractography. Nevertheless, immediate postoperative diffusion data is affected by blood degradation products and hemostatic materials that can impair a reliable tractography of both CST and OR and a delayed MRI could be affected by tumour progression, which justified the chosen technique. This technique does not allow for mapping and monitoring of visual-spatial neglect function. This function was not specifically considered in this study due to the lack of reproducibility of the cortico-cortical evoked potentials in the superior longitudinal fasciculus system and the limitations of the DTI for reconstruction of this system. Also, regardless the incidence of postoperative visuo-spatial neglect in this series (5/15), all patients were ambulatory at home 2 weeks after surgery [33].

Despite the above limitations, this study provides an integrated model with preoperative and intraoperative assessment of patients that are not eligible for or refused awake craniotomy for intra-axial non-dominant parietal lesions. Even though the more reliable technique that allows holistic patient-centred mapping and monitoring cannot be applied, we believe that an asleep multi-functional approach should be considered. This study shows that this dual-strip technique is safe and with reliable results that correlate with clinical outcomes.

## 6. Conclusions

This study demonstrates that real-time cortical and subcortical monitoring and mapping of the CST and optic radiations simultaneously is possible and accurate using two subdural electrode strips placed over the precentral gyrus and across the calcarine fissure. This feasibility study provides preliminary information about the way preoperative and intraoperative data can affect clinical outcomes and potentiate a deficit-sparing approach, as abnormal cortical excitability (particularly in the lower limbs), the cavity-to-tract (OR) distance and decrease of VEPs above 50% seems to be related with both motor and the visual outcome. 

## Figures and Tables

**Table 1:** Patient characteristics and clinical outcome.

Patient	Age Sex	Diagnosis	Use of US	Extent of Resection	Pre-Op Vision	Post-Op Vision	Pre-Op Motor Function	Post-Op Motor Function	Craniotomy Area (cm^2^)	Pre-Op Distance to CST (mm)	Post-Op Distance to CST (mm)	Pre-Op Distance to OR (mm)	Post-Op Distance to OR (mm)
1	74 M	Glioblastoma	Y	GTR	Hemianopia	No change	-	-	15.9	15	0	0	0
2	73 M	Glioblastoma	N	GTR	Hemianopia	No change	-	-	30.4	9.6	11.5	1	0
3	23 M	Pilocytic astrocytoma	Y	GTR	-	-	-	-	14.1	1	18.7	0	6.7
4	69 M	Glioblastoma *	N	GTR	Hemianopia	No change	-	Left hemiparesis	48.8	0	0	No OR	No OR
5	46 M	Glioblastoma	N	GTR	Hemianopia	No change	-	-	45.0	NA	NA	NA	NA
6	47 F	Glioblastoma	N	GTR	Hemianopia	No change	-	-	68.0	0	0	No OR	No OR
7	66 M	Glioblastoma	N	GTR	-	-	-	-	18.6	0	4.1	0	2
8	35 F	Glioblastoma	N	STR	-	-	-	-	15.0	1.5	3.9	1	2
9	55 F	Metastasis	N	GTR	Hemianopia	No change	-	-	7.6	18.1	22.6	0	1.9
10	59 M	Glioblastoma	Y	GTR	Hemianopia	No change	Left hemiparesis	Improvement—no deficit	22.5	5.2	3.7	1.3	0.3
11	66 F	Glioblastoma	Y	GTR	-	-	-	-	14.8	2	1.3	0	1.1
12	54 M	Glioblastoma *	N	GTR	Hemianopia	No change	Left foot paresis	Improvement—no deficit	91.9	NA	NA	NA	NA
13	57 M	Glioblastoma	N	STR	-	-	R hemiparesis (4/5)	Improvement—mild weakness (4+/5)	9.9	0.7	22.5	0.6	16.9
14	71 M	Glioblastoma	Y	GTR	-	-	-	-	35.3	1	0.8	2.2	8.3
15	49 M	Glioblastoma	Y	STR	Hemianopia	No change	Right hemiparesis	No change	42.4	0	2.5	0	0

US = ultrasound; Pre-op = pre-operative; post-op = post-operative; CST = corticospinal tracts; OR = optic radiation; M = male; F = female; * = recurrence; Y = yes; N = no; GTR = Gross total resection; STR = subtotal resection; L = left; R = right; NA = not assessed,- = no deficit.

## Supplementary Materials

The following are available online at https://www.mdpi.com/article/10.3390/jpm12091478/s1, Supplementary Methods and Illustrative Figure 3.

## References

[1] Bzdok D., Hartwigsen G., Reid A., Laird A.R., Fox P.T., Eickhoff S.B. (2016). Left inferior parietal lobe engagement in social cognition and language. Neurosci. Biobehav. Rev..

[2] Lavrador J.P., Ghimire P., Brogna C., Furlanetti L., Patel S., Gullan R., Ashkan K., Bhangoo R., Vergani F. (2021). Pre- and Intraoperative Mapping for Tumors in the Primary Motor Cortex: Decision-Making Process in Surgical Resection. J. Neurol. Surg. Part A Cent. Eur. Neurosurg..

[3] Husain M., Nachev P. (2007). Space and the parietal cortex. Trends Cogn. Sci..

[4] Freund H.J. (2003). Somatosensory and motor disturbances in patients with parietal lobe lesions. Adv. Neurol..

[5] Bisley J.W., Goldberg M.E. (2010). Attention, Intention, and Priority in the Parietal Lobe. Annu. Rev. Neurosci..

[6] Rolland A., Herbet G., Duffau H. (2018). Awake Surgery for Gliomas within the Right Inferior Parietal Lobule: New Insights into the Functional Connectivity Gained from Stimulation Mapping and Surgical Implications. World Neurosurg..

[7] Hejrati N., Spieler D., Samuel R., Regli L., Weyerbrock A., Surbeck W. (2019). Conscious Experience and Psychological Consequences of Awake Craniotomy. World Neurosurg..

[8] Nossek E., Matot I., Shahar T., Barzilai O., Rapoport Y., Gonen T., Sela G., Korn A., Hayat D., Ram Z. (2013). Failed awake craniotomy: A retrospective analysis in 424 patients undergoing craniotomy for brain tumor. J. Neurosurg..

[9] Hervey-Jumper S.L., Li J., Lau D., Molinaro A.M., Perry D.W., Meng L., Berger M.S. (2015). Awake craniotomy to maximize glioma resection: Methods and technical nuances over a 27-year period. J. Neurosurg..

[10] Yamamoto S., Masaki H., Kamata K., Nomura M., Ozaki M. (2018). A case of failed awake craniotomy due to progressive intraoperative hyponatremia. JA Clin. Rep..

[11] Matsuda A., Mizota T., Tanaka T., Segawa H., Fukada K. (2016). Difficult ventilation requiring emergency endotracheal intubation during awake craniotomy managed by laryngeal mask airway. Masui. Jpn. J. Anesthesiol..

[12] Rossi M., Nibali M.C., Viganò L., Puglisi G., Howells H., Gay L., Sciortino T., Leonetti A., Riva M., Fornia L. (2020). Resection of tumors within the primary motor cortex using high-frequency stimulation: Oncological and functional efficiency of this versatile approach based on clinical conditions. J. Neurosurg..

[13] Winston G.P., Yogarajah M., Symms M.R., McEvoy A.W., Micallef C., Duncan J.S. (2011). Diffusion tensor imaging tractography to visualize the relationship of the optic radiation to epileptogenic lesions prior to neurosurgery. Epilepsia.

[14] (2006). Guideline 5: Guidelines for Standard Electrode Position Nomenclature. J. Clin. Neurophysiol..

[15] Odom J.V., Bach M., Brigell M., Holder G.E., McCulloch D.L., Mizota A., Tormene A.P. (2016). ISCEV standard for clinical visual evoked potentials: (2016 update). Doc. Ophthalmol..

[16] Schucht P., SeiDel K., BecK J., MureK M., Jilch A., Wiest R., Fung C., Raabe A. (2014). Intraoperative monopolar mapping during 5-ALA–guided resections of glioblastomas adjacent to motor eloquent areas: Evaluation of resection rates and neurological outcome. Neurosurg. Focus..

[17] Seidel K., Beck J., Stieglitz L., Schucht P., Raabe A. (2013). The warning-sign hierarchy between quantitative subcortical motor mapping and continuous motor evoked potential monitoring during resection of supratentorial brain tumors. J. Neurosurg..

[18] Rosenstock T., Grittner U., Acker G., Schwarzer V., Kulchytska N., Vajkoczy P., Picht T. (2017). Risk stratification in motor area–related glioma surgery based on navigated transcranial magnetic stimulation data. J. Neurosurg..

[19] Neuloh G. (2010). Time to revisit VEP monitoring?. Acta Neurochir..

[20] Kodama K., Goto T., Sato A., Sakai K., Tanaka Y., Hongo K. (2010). Standard and limitation of intraoperative monitoring of the visual evoked potential. Acta Neurochir..

[21] Sarubbo S., De Benedictis A., Milani P., Paradiso B., Barbareschi M., Rozzanigo U., Colarusso E., Tugnoli V., Farneti M., Granieri E. (2015). The course and the anatomo-functional relationships of the optic radiation: A combined study with ‘post mortem’ dissections and ‘in vivo’ direct electrical mapping. J. Anat..

[22] Verst S.M., de Melo M.N., Caivano A.S., Fonseca U.S., Mathias L.R., Alves T.V. (2020). Awake surgery versus VEP in tumors of visual pathway: Case report. Interdiscip. Neurosurg..

[23] Chung S.B., Park C.W., Seo D.W., Kong D.S., Park S.K. (2012). Intraoperative visual evoked potential has no association with postoperative visual outcomes in transsphenoidal surgery. Acta Neurochir..

[24] Gomes D., Fonseca M., Garrotes M., Lima M.R., Mendonça M., Pereira M., Lourenço M., Oliveira E., Lavrador J.P. (2017). Corpus Callosum and Neglect Syndrome: Clinical Findings after Meningioma Removal and Anatomical Review. J. Neurosci. Rural Pract..

[25] Husar P., Berkes S., Götze A., Henning G., Plagwitz K.U. (2002). Improving SNR (signal to noise ratio) in multichannel EEG recording. Biomed. Tech. Biomed. Eng..

[26] Torres C.V., Pastor J., Rocío E., Sola R.G. (2012). Continuous monitoring of cortical visual evoked potentials by means of subdural electrodes in surgery on the posterior optic pathway. A case report and review of the literature. Revista de Neurología.

[27] Berro D.H., Herbet G., Duffau H. (2021). New insights into the anatomo-functional architecture of the right sagittal stratum and its surrounding pathways: An axonal electrostimulation mapping study. Brain Struct. Funct..

[28] Sollmann N., Zhang H., Kelm A., Schröder A., Meyer B., Pitkänen M., Julkunen P., Krieg S.M. (2020). Paired-pulse navigated TMS is more effective than single-pulse navigated TMS for mapping upper extremity muscles in brain tumor patients. Clin. Neurophysiol..

[29] Lavrador J.P., Gioti I., Hoppe S., Jung J., Patel S., Gullan R., Ashkan K., Bhangoo R., Vergani F. (2020). Altered Motor Excitability in Patients with Diffuse Gliomas Involving Motor Eloquent Areas: The Impact of Tumor Grading. Neurosurgery.

[30] Lavrador J.P., Gioti I., Hoppe S., Jung J., Patel S., Gullan R., Ashkan K., Bhangoo R., Vergani F. (2021). In Reply: Altered Motor Excitability in Patients with Diffuse Gliomas Involving Motor Eloquent Areas: The Impact of Tumor Grading. Neurosurgery.

[31] Fountas K.N., Smith J.R. (2007). Subdural Electrode-Associated Complications: A 20-Year Experience. Stereotact. Funct. Neurosurg..

[32] Joswig H., Lau J.C., Abdallat M., Parrent A.G., MacDougall K.W., McLachlan R.S., Burneo J.G., Steven D.A. (2020). Stereoelectroencephalography Versus Subdural Strip Electrode Implantations: Feasibility, Complications, and Outcomes in 500 Intracranial Monitoring Cases for Drug-Resistant Epilepsy. Neurosurgery.

[33] Galantucci S., Tartaglia M.C., Wilson S.M., Henry M.L., Filippi M., Agosta F., Dronkers N.F., Henry R.G., Ogar J.M., Miller B.L. (2011). White matter damage in primary progressive aphasias: A diffusion tensor tractography study. Brain.

