# Supplementary material for: Simultaneous Motor and Visual Intraoperative Neuromonitoring in Asleep Parietal Lobe Surgery: Dual Strip Technique
Source: J Pers Med. 2022 Sep 9;12(9):1478. doi: 10.3390/jpm12091478 (PMC9500827; doi:10.3390/jpm12091478)
Supplement: Supplementary file 1 [file jpm-12-01478-s001.zip › jpm-1888495-supplementary.pdf]

Supplementary Material

Methods

Monitoring and Mapping the Corticospinal Tract

After intubation of the patient, electrodes were placed in muscle groups of the hemibody contralateral to the tumour (face – *orbicularis oris* and tongue; upper limb – deltoid, *brachioradialis*, *flexor carpi ulnaris*, *first dorsal interossei*, *abductor digiti minimi*, *abductor pollicis brevis*; lower limb – quadriceps, *tibialis anterior* and *abductor hallucis*), to detect motor responses. Bilateral electrodes were placed in the extremities to act as controls.

Monitoring and Mapping Optic radiations

Whilst monitoring VEPs, a phase reversal of the VEPs were identified across the calcarine fissure. A bipolar fork-probe was used to stimulate subcortical tissue intra-operatively with a biphasic pulse form, 1-4Hz, and current dependent on proximity to the optic radiations (max 20mA). P2 and N3 peaks (International Society for Clinical Electrophysiology of Vision (ISCEV) standards) were measured at baseline, and during debulking[14] .

Tractography

The StealthViz® station (Medtronic) was used to anatomically delineate the Cortisospinal tract and Optic radiations. In order to visualise the ipsilateral CST, a region of interest (ROI) was placed over the pre-central gyrus and a second ROI on the midbrain. The FA start value of 0.18 was used along with a maximal directional change of 45°. With knowledge of the tract anatomy, manual dissection was used to remove spurious tracts. For the ipsilateral optic radiations, a ROI was placed over the lateral geniculate body and a second ROI over the visual cortex. Once again, the FA start value of 0.18 was used, with a maximal directional change of 60°. Fibres that were not following the anatomical pathway for these tracts were dissected appropriately.

nTMS (Motor Mapping)

nTMS uses a high-precision coil, neuronavigation, and appropriate software to deliver biphasic magnetic stimulation to the cortex. A single-pulse nTMS applied to the primary motor cortex, generates a muscle output that is recorded via continuous EMG.

nTMS was performed as a non-invasive adjunct for preoperative motor mapping. A T1 weighted post contrast MRI sequence for each patient was uploaded onto the Nexstim© (Helsinki, Finland) TMS hardware to enable accurate mapping of the motor cortex and collection of data on the resting motor threshold (RMT), latency, amplitude, interhemispheric resting motor threshold ratio (iRMTr), and the cortical excitability score (CES).

Continuous electromyography was used to monitor motor evoked potentials (MEPs) of the *abductor policis brevis* (APB), *first dorsal interossus* (FDI), and the *abductor digiti minimi* (ADM) in both upper limbs, as well as the *tibialis anterior* (TA) and *extensor hallucis longus* (EHL) in both lower limbs. Single pulse stimulation was applied at 7hz to both hemispheres at rest to identify the motor areas and ascertain the RMTs. Positive muscle responses were defined as MEPs greater than 50µV. Once determined, a final motor map was generated over the hemisphere of interest at 105% of the RMT.

The iRMTr was calculated as a ratio of the RMTs between the limbs in both hemispheres and was considered to be pathological if there was a difference of more than 10%. The Cortical Excitability Score (CES) was calculated and defined as the number of pathological iRMTs recorded: 0 (no pathological iRMTr present); 1 (only one pathological iRMTr present, either for the upper or lower limb); and 2 (when both upper and lower limb demonstrated a pathological iRMTr)

The motor maps generated were exported as DICOM files and used for intraoperative augmented reality and navigation.

Illustrative Figure 3

Pre-processed diffusion-weighted MRI data were retrieved for a young adult male from the Human Connectome Project (www.humanconnectome.org, accessed on 8/6/2022)[34]. Diffusion tensor modelling and tractography were computed in StarTrack (www.mr-startrack.com, accessed on 2/07/2022)[35] according to the following criteria: minimum fractional anisotropy threshold = 0.2; step size = 0.5 mm; maximum angle threshold = 30°. The optic radiations and corticospinal tract were manually dissected in TrackVis

(www.trackvis.org, accessed on 3/7/2022)[36]. The CST was dissected using an axial waypoint ROI at the level of the pons and another to intersect terminations in sensorimotor cortex. The optic radiation was dissected using a termination ROI at the level of the lateral geniculate nucleus and a coronal waypoint ROI at the level of the occipital lobe. The dissected tracts were aligned to the MNI152 brain template for final display within the brain surface in SurfIce (www.nitrc.org/projects/surfice, accessed on 1/7/2022)[37]. The locations of each patient's response points for the CST and OR were manually estimated on the MNI152 T1-weighted image, and the resulting coordinates were imported into the final display (Figure 3) for illustrative purposes.

Supplemental References

34. Human Connectome Project. Available online: http://www.humanconnectome.org. (accessed on 8/6/2022).

35. StarTrack. Available online: http://www.mr-startrack.com (accessed on 2/7/2022).

36. TrackVis. Available online: http://www.trackvis.org (accessed on 3/7/2022).

37. SurfIce. Available online: http://www.nitrc.org/projects/surfice (accessed on 1/7/2022)).
